# Structure and Characterizations of Different Hydrophobically Modified Phytoglycogen Nanoparticles

**DOI:** 10.3390/foods14081351

**Published:** 2025-04-14

**Authors:** Huixuan Wang, Ming Miao

**Affiliations:** State Key Laboratory of Food Science & Technology, Jiangnan University, 1800 Lihu Avenue, Wuxi 214122, China; 18767950276@163.com

**Keywords:** phytoglycogen, esterification, carbon chain length, structure characterization

## Abstract

This study aimed to chemically modify natural phytoglycogen (PG) nanoparticles with three different alkenyl succinic anhydrides to characterize their physicochemical properties and assess the feasibility of using the modified PG nanoparticles (octenylsuccinic anhydride, (OSA)-PG; dodecenylsuccinic anhydride, (DDSA)-PG; and octadecenylsuccinic anhydride, (ODSA)-PG) as carriers for hydrophobic bioactive compounds. The results showed that under the same addition level, the degree of substitution (DS) of modified PG decreased with the extension of the carbon chain in different alkenyl succinic anhydrides, while for the same alkenyl succinic anhydride, the DS increased with higher additive amounts. The higher the DS of modified PG, the more pronounced the effect of pH on the zeta potential. Both OSA-PG and DDSA-PG demonstrated excellent solubility and stability in aqueous environments, whereas ODSA-PG exhibited markedly reduced solubility and stability. Compared to native PG, different hydrophobically modified PG exhibited improved rheological and digestion properties. Among them, DDSA-PG exhibited higher shear stability than OSA-PG, but OSA-PG was more resistant to enzyme degradation. The findings of this study indicate that PG modified with different carbon chain lengths of hydrophobic anhydride groups has great application potential and offers a theoretical basis for the construction of pH-responsive nanocarriers and lipopolysaccharide transport carriers.

## 1. Introduction

Phytoglycogen (PG) is a nanosized glucan derived from plants, primarily sourced from the kernels of the corn mutant sugar-1 (*su1*) [1]. The particle size of PG ranges from 30 to 100 nm, with an average chain length of 11 to 12 glucose units and a branching density of 8% to 9% [2]. PG exhibits a highly branched spherical structure, with its density increasing from 950 to 1200 g/mol·nm^3^ from the center to the outer surface of the nanoparticle [3]. These structural features contribute to the distinctive properties of PG, including excellent water retention, low viscosity, and outstanding stability. Additionally, PG can be modified through either chemical or enzymatic methods to enhance its performance. Enzymes such as β-amylase, amylosucrase, and phosphorylase are used to alter its digestive and physicochemical properties [4,5,6]. While enzyme modification is considered an environmentally friendly approach, it is still limited by its scalability and cost-effectiveness.

In contrast, chemical modification of PG is relatively simple, cost-effective, and efficient. OSA modification refers to the reaction between the succinic anhydride group in OSA molecules and the hydroxyl groups in starch molecules, which partially replaces the hydroxyl groups in starch and forms ester bonds. Through this esterification reaction, hydrophobic succinate ester groups are introduced into the starch molecules, thereby imparting excellent amphiphilicity and good interfacial properties to the starch [7]. OSA esterification is typically carried out in an aqueous environment. Compared to other modification methods, OSA modification does not require the use of harmful organic solvents, and the reaction conditions are mild (temperature around 30–35 °C, pH around 8.5), with high reaction yields [8]. Additionally, compared to other modification methods such as hydroxypropylation, acetylation, or cross-linking, OSA modification significantly improves the levels of SDS and RS, resulting in high SDS functional fibers [9]. Moreover, OSA-modified starch is generally considered safe and has been approved by the U.S. Food and Drug Administration (FDA) for use as a food additive. Furthermore, OSA modification significantly enhances the solubility of hydrophobic substances such as curcumin, lutein, and β-carotene [10,11].

DDSA and ODSA are esterifying agents with structures similar to OSA, but with 12- and 18-carbon chains, respectively. Modified starches with these longer alkenyl chains are commonly used as paper sizing agents. Compared to OSA, DDSA and ODSA exhibit increased hydrophobicity at the same DS. While the FDA has approved a modification level of <3% for OSA in foods (DS 0.023), DDSA and ODSA may provide similar effects at lower DS, presenting promising alternatives for food applications. Recent studies have demonstrated the potential of DDSA and ODSA in the food industry. Kokubun et al. synthesized OSA- and DDSA-modified inulin derivatives with varying DS [12]. Both dye solubilization and surface tension measurements revealed that the DDSA-modified inulin exhibited a significantly lower critical micelle concentration (CMC) than its OSA-modified counterpart, demonstrating enhanced micellization efficiency at lower concentrations. β-carotene loading experiments indicated that DDSA-modified inulin, particularly the highly substituted DDSA variant, displayed greater solubilization efficacy, potentially attributable to its more stable hydrophobic core structure. In a related study, Zhang developed oleyl-modified γ-cyclodextrin metal–organic frameworks (OCMs) using ODSA for the stabilization of Pickering emulsions [13]. The results demonstrated that the elongated hydrophobic chain of ODSA facilitated stronger interfacial adsorption, yielding Pickering emulsions with enhanced stability. When applied as edible coatings, the ODSA-modified OCMs effectively reduced postharvest deterioration, with treated strawberries showing only 15.6% weight loss after 9 days of storage.

While conventional modified starches have been extensively studied, the unique nanostructure of PG offers distinct advantages for carrier applications. Unlike linear or moderately branched starch derivatives, PG’s three-dimensional dendritic architecture with a gradient density distribution provides both high surface accessibility and an internal scaffold structure [14]. This intrinsic nanostructure allows simultaneous modification of surface hydroxyl groups and encapsulation within the particle core, a feature unattainable in conventional starch granules with crystalline domains. Xie et al. [15] developed octenylsuccinate hydroxypropyl PG (OHPP) and used it to load active pharmaceutical ingredients (APIs), which increased the solubility of the API by up to 1755 times, thereby demonstrating the potential of modified PG for loading applications.

In addition, the existing literature has mainly focused on enzyme modification or short-chain chemical modifications of PG. However, the effects of modifying PG with different alkenyl succinic anhydrides (OSA, DDSA, ODSA) and how these modifications influence key properties, such as molecular aggregation and enzymatic resistance, remain underexplored. This research aims to fill this gap by systematically analyzing PG modified with three different esterifying agents (OSA, DDSA, ODSA) in terms of their degree of substitution (DS), solubility, rheological properties, and enzymatic degradation behavior. Specifically, this study will explore how the elongation of the hydrophobic chain impacts the functional performance of PG-based carriers. The results will provide novel insights into the design of PG-based carriers for drug delivery systems, with potential applications in food and pharmaceutical industries. By revealing the correlation between chain length and functional properties, this study offers both theoretical guidance and practical insights for creating advanced delivery systems.

## 2. Materials and Methods

### 2.1. Materials

Sweet corn was obtained from the Chinese Academy of Agricultural Sciences (Guangzhou, China). OSA (≥95% purity) and amyloglucosidase (260 U/mL, EC 3.2.1.3) were purchased from Aladdin Biochemical Technology Co. (Shanghai, China). DDSA (≥95% purity) and ODSA (≥93% purity) were purchased from Macklin Biochemical Technology Co. (Shanghai, China). α-Amylase (75,000 U/g, EC 3.2.1.1) was purchased from Megazyme International Limited (Wicklow, Ireland). All other chemicals used were of analytical grade.

### 2.2. Extraction of Phytoglycogen

Fresh sweet corn (50 g) was soaked in 250 g deionized water at 4 °C for 12 h and then ground using a pulping machine. The mixture was sieved through a 300-mesh filter. The supernatant was adjusted to a pH of 4.9 using acetic acid. After a 2 h incubation at 4 °C, insoluble proteins were removed by centrifugation (6000 rpm, 30 min). The pH was then neutralized to 7.0 with 1 M sodium carbonate solution. The solution was heated in a water bath for 30 min, followed by centrifugation to eliminate remaining proteins. Finally, three volumes of ethanol were added to induce the precipitation of PG. The solids were dried for further use.

### 2.3. Alkenyl Succinic Anhydride Modification

A 30% (*w/v*) PG dispersion was prepared and stirred overnight to ensure uniformity [16]. OSA, DDSA, and ODSA were dissolved in isopropanol at a 1:5 (*v/v*) dilution ratio and added dropwise to the PG dispersion over 2 h to achieve an anhydride content of 3% or 9%. The reaction was conducted at 35 °C and maintained pH 8.5 with NaOH solution. After continuing the reaction for an additional 6 h, the pH was adjusted to 6.5 with HCl to terminate the reaction. Subsequently, 3 volumes of ethanol were introduced to induce the precipitation of substituted PG. The samples were labeled as 3%OS-PG, 9%OS-PG, 3%DDS-PG, 9%DDS-PG, 3%ODS-PG, and 9%ODS-PG, where the numbers indicate the final concentration of the anhydride reagent.

### 2.4. Determination of DS of Modified PG

The modified PG samples (0.5 g) were acidified by adding 3 mL of 2.5 M HCl and incubating for 30 min [17]. Then, 10 mL of 90% isopropanol was added to the suspension, which was then centrifuged at 3000 rpm for 10 min to separate the precipitated complexes. The precipitate was then washed with 90% isopropanol until no white AgCl precipitate formed upon addition of 0.1 M AgNO_3_ solution to the wash liquid. Next, the washed precipitate was dissolved in 30 mL of deionized water and heated in a boiling water bath for 30 min. Immediately after heating, the solution was titrated with 0.1 M NaOH, using native PG as a blank and phenolphthalein as the end-point indicator. Finally, DS was calculated using the following formula:

$$\mathrm{DS}=\frac{162\times (C\times V)/W}{1{-M}_{i}(C\times V)/W}$$

where C was the concentration of NaOH; V was the volume of NaOH; W was the dry weight of PG, 162 was the molar mass of glucose residue and M_i_ was the molar mass of modifying agent.

### 2.5. FT-IR

The samples were analyzed using a Nicolet IS10 FTIR spectrophotometer (Thermo Scientific, Waltham, MA, USA) fitted with an ATR accessory. The spectral range was scanned from 400 to 4000 cm^−1^ with a resolution of 4 cm^−1^, performing 32 scans in total [18].

### 2.6. Zeta-Potential and Particle Size Distribution

PG samples (0.1%, *w/v*) were suspended in deionized water. The particle size and zeta potential were measured using a Zetasizer Nano ZS (Malvern Instruments Ltd., Malvern, Worcestershire, UK) at room temperature. Another set of PG samples were completely dissolved and adjusted to pH 3, 5, 7, 9, 11, and 12 using 2.5 M HCl or 2.5 M NaOH solution. After stabilizing the samples at 4 °C for 24 h, the zeta-potential of each sample was measured.

### 2.7. Turbidity Analysis

PG samples (0.5%, *w/v*) were dispersed in deionized water and vortexed to achieve full dissolution. The samples were then centrifuged at 6000 rpm for 10 min. A 721G Visible Spectrophotometer (Shanghai Yidian Instrument Co., Ltd., Shanghai, China) was used to measure the transmittance (T%) at 620 nm, with deionized water serving as the blank.

### 2.8. Water Solubility of Modified PG

To assess solubility, 2 g of PG and modified PG were dissolved in 20 mL of deionized water and stirred for 15 min. The mixture was then centrifuged at 6000 rpm for 10 min, and the supernatant was transferred to weighing bottles. Finally, the samples were dried at 105 °C until a constant weight was achieved [19]. The water solubility (WS) was calculated using the following formula:

$$\mathrm{WS}=\frac{\mathrm{Dry}\;\mathrm{weight}\;\mathrm{of}\;\mathrm{solubilized}\;\mathrm{PG}}{\mathrm{Dry}\;\mathrm{weight}\;\mathrm{of}\;\mathrm{PG}}$$

### 2.9. Molecular Weight Distribution Profiles

The weight-average molar mass (Mw) and the z-root mean square radius of gyration (Rz) of the samples were measured using a high-performance size exclusion chromatography (HPSEC) system coupled with multi-angle laser light scattering (MALLS) and refractive index (RI) detectors. (Wyatt Technology, Santa Barbara, CA, USA). The samples were first dissolved in deionized water to achieve a concentration of 0.5% (*w/v*), then filtered through a 0.22 μm cellulose filter prior to injection. The chromatographic system consisted of two Shodex OH-pak columns (SB-806 HQ and SB-804 HQ) connected in series, with the column temperature maintained at 50 °C. A mobile phase consisting of 0.2 M sodium nitrate and 0.05% antimicrobial agent was employed, with a flow rate set to 0.6 mL/min. Data analysis was performed using ASTRA software (Version 5.3.4, Wyatt Technology).

### 2.10. Flow Behavior Analysis

The rheological behavior of PG nanoparticle suspensions (10%, *w/v*) was measured using a Discovery HR-3 Rheometer (TA Instruments, New Castle, DE, USA). The plate geometry had a diameter of 40 mm, with a gap height set at 1 mm [20]. A circulating bath along with a controlled Peltier system was employed to maintain a constant temperature of 25 °C. The flow sweep test was conducted by continuously shearing the samples from 1 to 100 s^−1^. The data obtained from the test, including shear stress and shear rate, were fitted to the Power Law and Herschel–Bulkley models, respectively.

$$\sigma =K\gamma^{n}$$

$$\sigma =\sigma_{0}+K\gamma^{n}$$

where σ represented the shear stress, Pa; K denoted the consistency index, Pa s^n^; γ was the shear rate, s^−1^; n indicated the flow behavior index.

### 2.11. In Vitro Digestion

The digestibility of the samples was assessed following the method of Englyst et al. with some modification [21]. The enzyme solution was prepared by dissolving pancreatic α-amylase in deionized water to a concentration of 580 U/mL, then adding amyloglucosidase to a concentration of 30 U/mL. The samples (100 mg) were dissolved in 5 mL of sodium acetate buffer (0.2 M, pH 5.2) and 3 mL of deionized water. The experiment was performed in a water bath shaker set at 37 °C. After allowing the samples to stabilize for 10 min, 2 mL of enzyme solution was added to initiate the reaction. Aliquots (0.05 mL) were withdrawn at certain time point and were mixed with 0.45 mL of ethanol. After centrifugation, the amount of glucose in the supernatant was quantified using the DNS method. The amounts of rapidly digestible starch (RDS), slowly digestible starch (SDS), and resistant starch (RS) were calculated based on the following formulas:

$$RDS\left(\%\right)=\left[\left(G_{20}-FG\right)∕TS\right]\times 0.9\times 100$$

$$SDS\left(\%\right)=\left[\left(G_{120}-G_{20}\right)∕TS\right]\times 0.9\times 100$$

$$RS\left(\%\right)=\left[\left(TS-RDS-SDS\right)∕TS\right]\times 100$$

where G_20_ referred to the glucose released after 20 min, G_120_ represented the glucose released after 120 min, FG denoted free glucose, and TS stood for total starch.

### 2.12. Thermogravimetric Analysis

The thermogravimetric analysis was performed using the TGA3 thermogravimetric analyzer (Mettler Toledo Instruments Co., Greifensee, Zurich, Switzerland). The temperature was increased from 30 to 550 °C at a rate of 10 °C/min, while maintaining a nitrogen flow rate of 20 mL/min. The differential thermogravimetric (DGT) curve was obtained by calculating the first derivative of the TGA data using ORIGIN 2018 software (OriginLab Inc., Northampton, MA, USA).

### 2.13. Statistical Analysis

All experiments were conducted in triplicate, and the results are presented as mean values ± standard deviations. The mean values were analyzed using Tukey’s multiple comparisons and one-way analysis of variance (ANOVA) via SPSS (version 26.0). A *p*-value of less than 0.05 was considered to indicate a significant difference.

## 3. Results and Discussion

### 3.1. Preparation of Modified PG

#### 3.1.1. FT-IR

**Figure 1:**
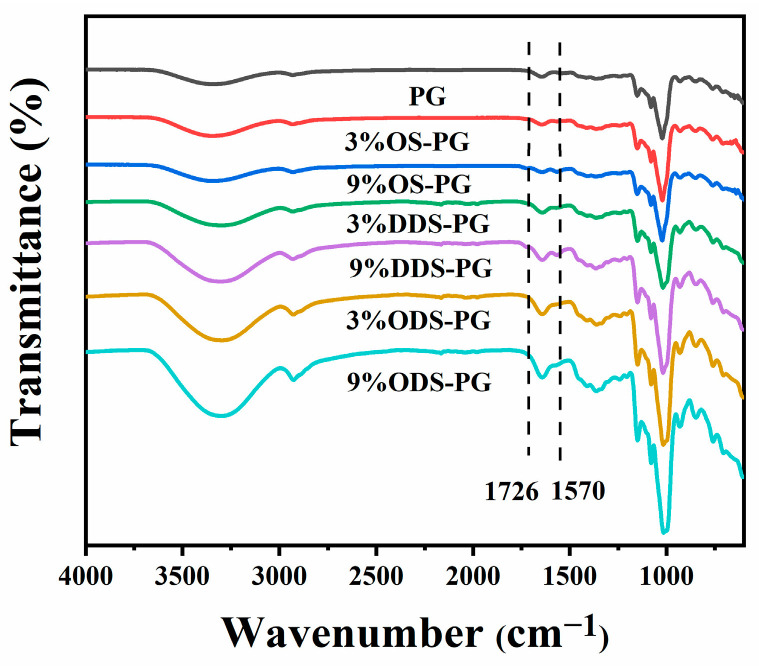
FT-IR spectra of native phytoglycogen (PG) and different modified PGs.

Figure 1 presents the FT-IR spectra of natural PG and PG modified with various succinic anhydrides. The carboxylic acid groups in alkenyl succinic anhydride can be grafted onto PG molecules through an esterification reaction with the hydroxyl (-OH) groups of PG. Compared to natural PG, alkenyl succinic anhydride-modified PG displayed two new absorption peaks at 1726 cm^−1^ and 1570 cm^−1^. These two peaks corresponded to the stretching vibrations of the carboxylate group (RCOO⁻) and the ester carbonyl group (C=O) [22], indicating that succinic anhydride had been successfully grafted onto PG. When PG was modified with equal amounts of succinic anhydride of different chain lengths, the absorption peak intensities of RCOO⁻ and C=O decreased as the chain length of the succinic anhydride increased. This likely suggests that longer chains may cause steric effects or hinder the reaction, resulting in fewer esterification sites. In contrast, when the same chain length of succinic anhydride was used for modification, the peak intensities of both functional groups increased as the amount of succinic anhydride added increased, indicating an increase in the esterification degree. A similar phenomenon was observed in experiments involving agar modification [22,23]. In addition to the appearance of new absorption peaks, the hydroxyl absorption peak at 3400 cm^−1^ shifted toward higher wavenumbers and became narrower. This phenomenon suggested a reduction in hydrogen bonding within the agar molecules during the esterification process, further confirming the occurrence of the esterification reaction.

#### 3.1.2. The DS of Modified PG

DS is a crucial index for assessing the extent of esterification in starch. The DS values for PG modified with various alkenyl succinic anhydrides are presented in Table 1. On one hand, the DS of modified PG decreased significantly with increasing carbon chain length of alkenyl succinic anhydrides (*p* < 0.05). Compared to OS-PG, DS values for 3% DDS-PG and 3% ODS-PG decreased by 30.06% and 50.98%, respectively. Similarly, at 9% anhydride addition, DDS-PG and ODS-PG exhibited 35.41% and 76.85% lower DS than OS-PG. This trend is attributed to the higher molecular weight and lower water solubility of long-chain anhydrides (DDSA, ODSA) [24], which limit their effective interaction with PG.

On the other hand, the DS of 9%OS-PG, 9%DDS-PG, and 9%ODS-PG were 2.82, 2.60, and 1.33 times that of 3%OS-PG, 3%DDS-PG, and 3%ODS-PG, respectively. The increase in DS of OS-PG was more significant compared to DDS-PG and ODS-PG when the addition increased from 3% to 9%. The diminished DS gain in long-chain derivatives stems from steric hindrance and hydrophobic aggregation, as bulky substituents shield the reactive hydroxyl groups at the surface and branch points of PG.

### 3.2. Structure of Modified PG

#### 3.2.1. Molecular Weight Distribution Profiles of PG and Modified PG

Table 1 indicates that the molecular weight of PG typically decreased after modification with three different alkenyl succinic anhydrides. The esterification reaction typically occurs in an aqueous solution under warm, mildly alkaline conditions. The alkaline environment, along with agitation, may cause degradation of PG molecules, resulting in a decrease in their Mw. However, an increase in Mw was observed for OS-PG. This may be due to its high DS, which compensated for the degradation of PG. At higher anhydride concentrations, the molecular weight further decreased, likely because excessive alkali addition to maintain pH balance exacerbated the degradation of PG [25]. Furthermore, the molecular weight disparity between samples increased as the chain length of the anhydrides grew. This phenomenon could be explained by the limited enhancement of DS with long-chain anhydrides (e.g., ODS-PG) compared to short-chain ones (e.g., OS-PG), due to steric hindrance and reduced solubility. The hydrophobic modification increased the Rz of PG. On the one hand, despite a slight reduction in Mw, it remained at the 10^7^ g/mol level, indicating that the core structure of PG was preserved. In addition, the introduced alkyl chains likely extended the spatial conformation of PG molecules, leading to an increase in Rz. Moreover, the enhancement of Rz became more pronounced with higher anhydride content, suggesting that increased substitution density further expanded the molecular dimensions.

#### 3.2.2. Solution Properties of PG and Modified PG

DLS measurements showed that the particle size of modified PG increased compared to natural PG. For 3%OS-PG and 9%OS-PG, the increases were 6.66% and 8.86%, respectively; for 3%DDS-PG and 9%DDS-PG, they were 7.84% and 14.38%, respectively. The most significant size increases were observed in 3%ODS-PG and 9%ODS-PG, with increases of 19.06% and 27.33%, likely due to enhanced hydrophobic interactions from the longer ODSA chains, which promoted aggregation. The discrepancy between Rz and DLS may be due to DLS measuring the hydrodynamic diameter of hydrated particles, including both single chains and aggregates stabilized by hydrophobic interactions.

**Figure 2:**
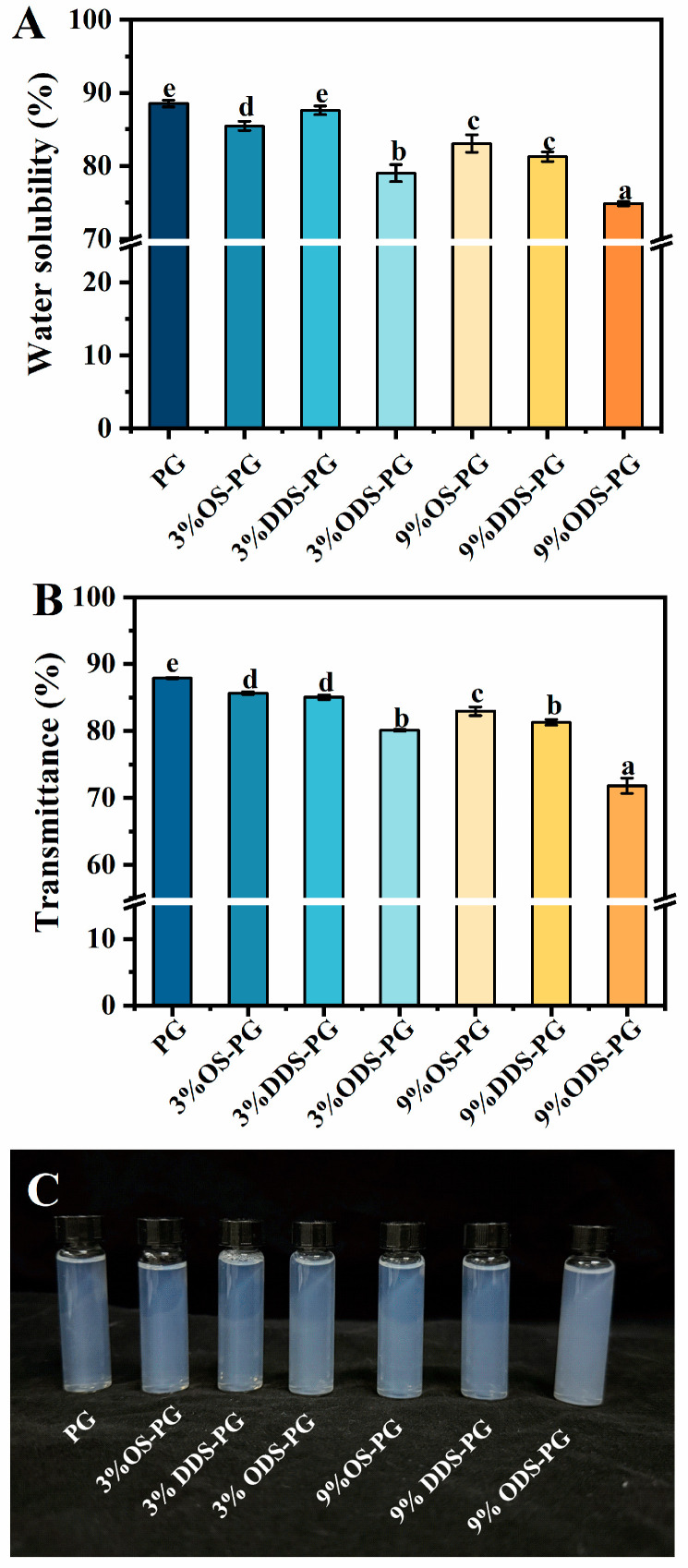
Solution properties of samples: (**A**) solubility of different samples; (**B**) transmittance of samples at 620 nm; (**C**) digital picture of turbidity of samples. Different letters above the bars indicate significant differences (*p* < 0.05). Values are expressed as the mean ± SD (*n* = 3).

To further assess the impact of alkenyl succinic anhydride modification on the aggregation behavior of the modified PG, the turbidity of the samples was tested, as shown in Figure 2. When 3% and 9% alkenyl succinic anhydride with varying carbon chain lengths were added, the light transmittance of OS-PG, DDS-PG, and ODS-PG decreased compared to that of the original PG. The light transmittance of ODS-PG was reduced by 8.87% and 18.32%, respectively. The decrease in light transmittance was likely due to the formation of aggregates in alkenyl succinic anhydride-modified PG, which was consistent with the DLS results.

The water solubility of PG was 87.3%. PG had a highly branched and unique nanoscale structure, which contributed to its high solubility [2,19]. After modification, the solubility of 3%OS-PG and 3%DDS-PG decreased slightly. 3%DDS-PG showed higher solubility than 3%OS-PG, likely due to its lower DS, which partially offset DDSA’s higher intrinsic hydrophobicity. As the amount of anhydride added increased, the solubility of the modified PG decreased significantly. Despite having a lower DS, 9%DDS-PG exhibited lower solubility than 9%OS-PG, which may be due to the longer carbon chains of substituted DDSA, leading to increased hydrophobicity. ODS-PG had the lowest solubility, regardless of the amount of anhydride added, due to the longest carbon chain in the substituted anhydride.

**Figure 3:**
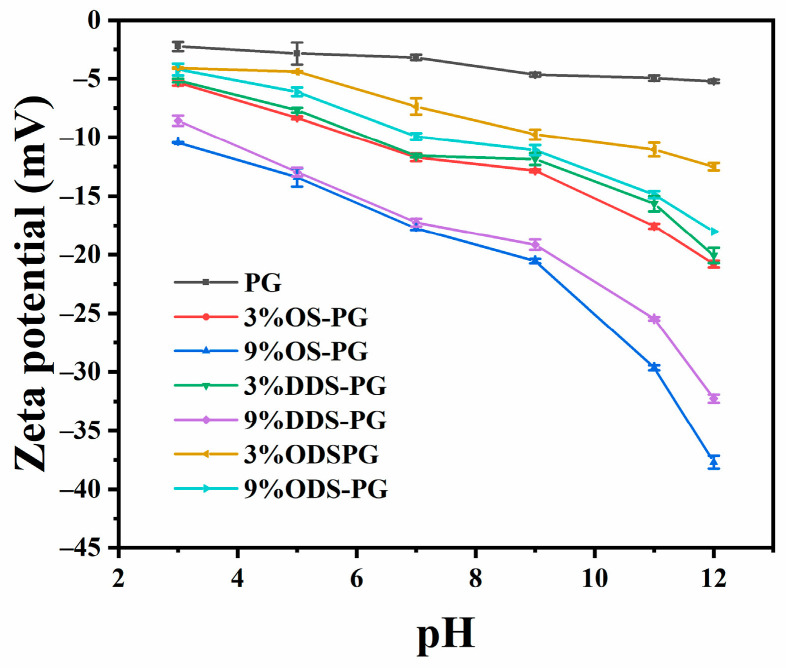
Zeta potential of native phytoglycogen (PG) and modified PGs under different pH. Values are expressed as the mean ± SD (*n* = 3).

As shown in Table 1, the absolute value of the zeta potential increased for all modified samples compared to natural PG. Additionally, when the same type of alkenyl succinic anhydride was used, the absolute zeta potential of the modified PG exhibited a positive correlation with the amount of anhydride added. This indicated that more carboxyl groups were grafted onto the PG molecule, leading to a higher surface charge density, which was consistent with previous studies [26]. The absolute value of the zeta potential for ODS-PG was lower compared to OS-PG and DDS-PG, possibly due to its lower DS. A low zeta potential indicated the instability of the samples. As illustrated in Figure 3, the influence of pH on the zeta potential of modified PG was examined. The zeta potential of natural PG showed little change across the pH range of 3–12. However, for PG modified with different alkenyl succinic anhydrides, the absolute zeta potential increased significantly within this pH range, indicating that the deprotonation of carboxyl groups in PG molecules increased with the pH. This pH sensitivity was useful for constructing pH-responsive nanocarriers using modified PG.

In summary, the performance of modified PG nanoparticles is primarily regulated through the interaction between chain length and DS. Chain length directly affects DS, with short chains resulting in higher DS and reduced steric hindrance due to their smaller molecular size and better water solubility, while long chains lead to lower DS because of their bulky size and poor solubility. The relationship between DS and chain length also governs molecular aggregation, as higher DS introduces more charged carboxyl groups, increasing surface charge density and stabilizing the dispersion through electrostatic repulsion, which mitigates aggregation driven by hydrophobicity. In contrast, lower DS allows hydrophobic interactions to dominate, resulting in enhanced aggregation and larger particle size. These variations also affect solubility, with long chains reducing solubility and promoting aggregation, while short chains maintain higher solubility and suppress aggregation. Overall, chain length and DS directly influence hydrophobic interactions, charge density, and aggregation behavior in PG nanoparticles.

Considering factors such as solubility and stability, OSA-PG and DDSA-PG were selected for the next experiments.

### 3.3. Physiochemical Properties of Modified PG

#### 3.3.1. Rheological Behavior of PG and Modified PG

**Figure 4:**
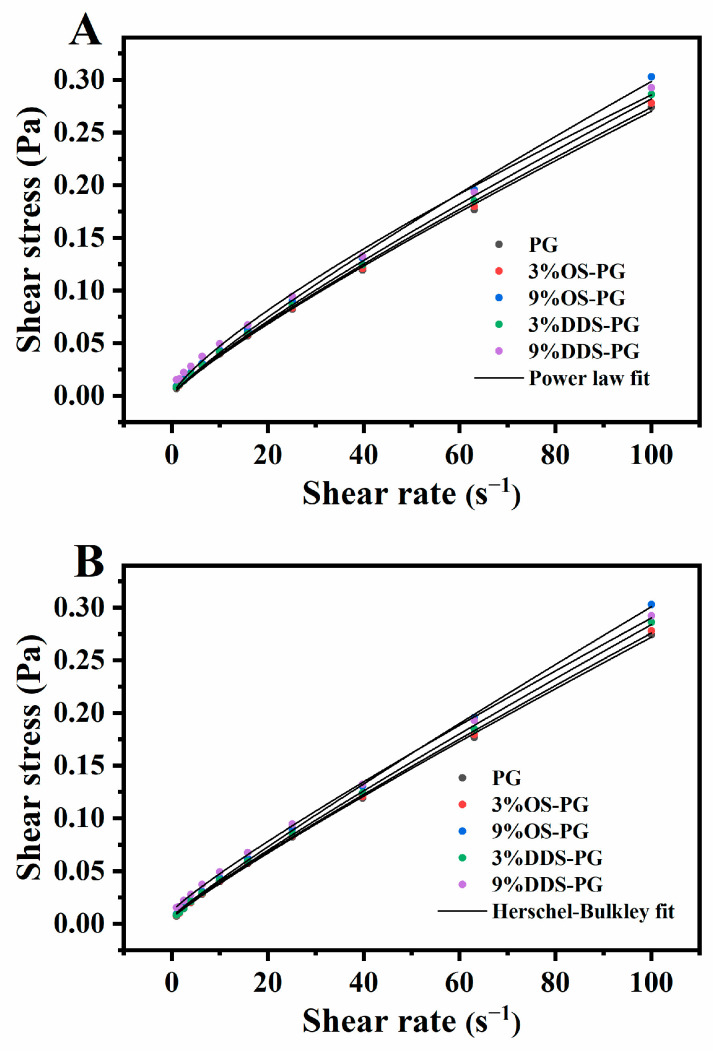
Shear stress–shear rate plots for modified phytoglycogens, fitted using two different methods: (**A**) Power Law fit; (**B**) Herschel–Bulkley fit.

Figure 4 shows the plots of shear stress versus shear rate for different samples. The data obtained from the experiment for all samples fit well with both the Power Law and Herschel–Bulkley models. As indicated in Table 2, the Herschel–Bulkley model (R^2^ = 0.999) fitted better compared to the Power Law model (R^2^ = 0.995~0.999). According to the Herschel–Bulkley model, the value of n reflects the degree of non-Newtonian behavior [27]. In this experiment, n for all samples was less than 1, indicating that all modified PG solutions were pseudo-plastic fluids. K represents the apparent viscosity of the examined suspensions [1]. At concentrations below 20% (*w/v*), the intrinsic shear viscosity of the PG dispersions is relatively low (<70 mPa·s), suggesting that they exhibit characteristics similar to rigid, compact spheres [28]. In previous research, Ye et al. [1] reported that at a concentration of 5%, the K values for OSA-modified PG and acid-treated waxy maize starch were 0.0035 and 0.1872, respectively. This indicates the low viscosity of PG and modified PG, which is advantageous for drug loading. In our study, the viscosity of modified PG increased compared to native PG. This was attributed to the increased hydrophobic moieties in modified PG, which might contribute to heightened repulsive forces between groups, resulting in increased viscosity [20]. At the same addition level, DDS-PG showed higher viscosity than OS-PG, which was contrary to Xue et al. [29]. This may be because longer carbon chains of DDSA cause more entanglements in the paste, leading to higher viscosity [30]. According to Midmore, σ_0_ indicates the ability of the network to resist sedimentation or creaming [31]. DDS-PG showed a higher σ_0_ compared to OS-PG, indicating its higher stability against shearing, which was important in fluid transportation.

#### 3.3.2. In Vitro Digestion of PG and Modified PG

**Figure 5:**
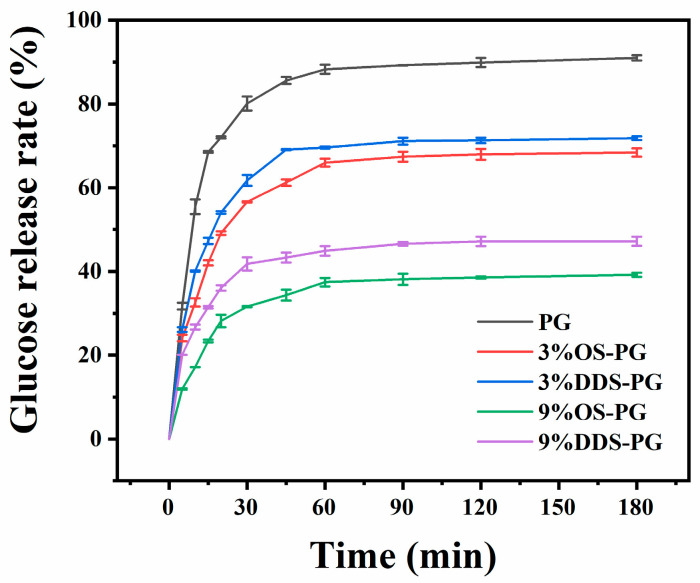
Hydrolysis of native phytoglycogen (PG) and modified PGs by amyloglucosidase and α-amylase over 180 min. Values are expressed as the mean ± SD (*n* = 3).

Figure 5 shows the hydrolysis profiles of both native PG and modified PG over a period of 180 min. The glucose release rate of native PG reached 80.1% within the first 30 min and steadily increased to 90.1% by the end of 180 min. Modified PG exhibited a similar yet slower trend. Both the type of anhydride and the addition amount significantly influenced glucose release. For PG modified with 3% OS-PG and DDS-PG, the glucose release rate at 30 min decreased by 29.3% and 22.9%, respectively, while the release rate at 180 min decreased by 24.7% and 21.0%, respectively. Additionally, increasing the addition amount resulted in a pronounced reduction in glucose release. When the addition amount was increased from 3% to 9%, the glucose release rate at 30 min for OS-PG and DDS-PG decreased by 60.5% and 47.81%, respectively, and at 180 min, the release rates decreased by 56.9% for OS-PG and 48.14% for DDS-PG.

Based on the rate and degree of starch digestion, starch was categorized into RDS, SDS, and RS [21]. As shown in Table 3, the RDS, SDS, and RS content in PG were 69.5%, 17.89%, and 12.61%, respectively. According to Miao et al., the high branching density of PG made it has some kind of resistant to amylolytic enzymes [1]. After esterification, the RDS content for 9%OS-PG and 9%DDS-PG decreased by 63.4% and 51.8%, respectively, while the RS content for 9%OS-PG and 9%DDS-PG increased by 4.1 times and 3.4 times. This result indicated that esterified PG had a considerable inhibitory effect to degradation, perhaps due to the bulky anhydride group that created physical barriers or hydrophobic microenvironments which delayed the enzyme’s access to the substrate [32]. OS-PG exhibited a more significant reduction in PG digestibility compared to DDS-PG, which could be due to a higher number of anhydride groups being grafted onto PG.

#### 3.3.3. Thermal Properties of PG and Modified PG

**Figure 6:**
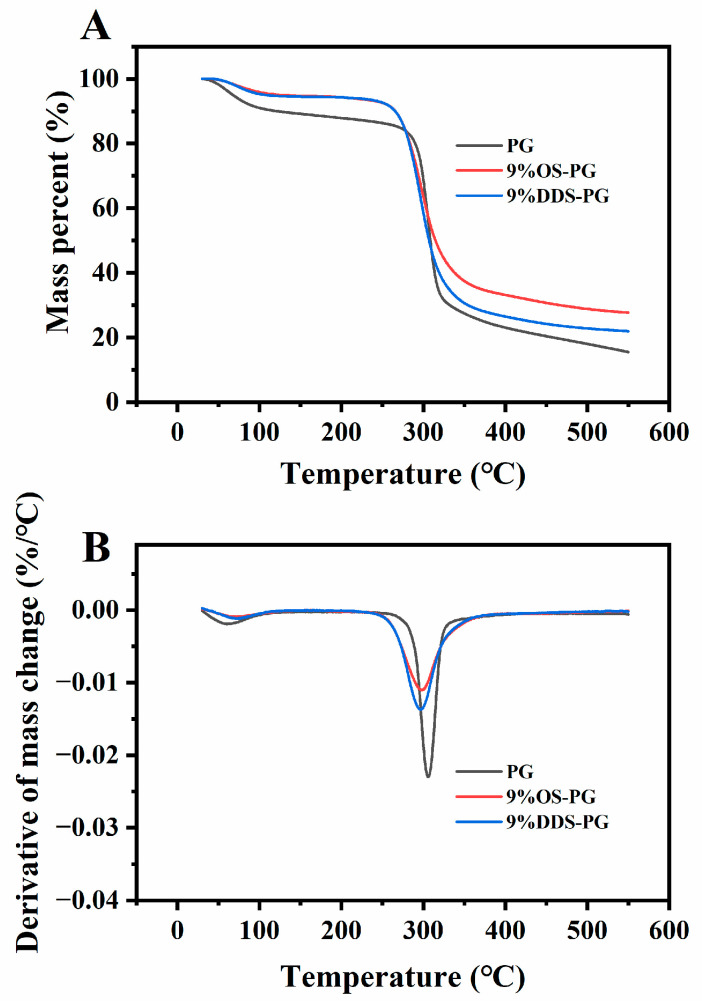
Thermogravimetric curves of phytoglycogen (PG) and modified PGs: (**A**) TG; (**B**) DTG.

TGA and DTG curves are presented in Figure 6. According to previous research, the thermal degradation of PG includes three main stages: evaporation of water (mass loss of 8.19%), depolymerization of materials (mass loss of 58%), and formation of graphitic carbon (mass loss of 16.93%). In the end, approximately 16.25% of the solid residue remains [20]. In our experiment, the weight loss rates in the three stages were 7.6%, 60.23%, and 16.65%, respectively, with a final residue of 15.49%, which was similar to the reported PG decomposition results. For modified PG, the weight loss in the first stage was less than that of PG, possibly because the addition of hydrophobic alkenyl chains reduced their tendency to interact with water molecules [33]. Additionally, the modified PG in the second stage exhibited a lower initial decomposition temperature compared to native PG. This reduction was attributed to the rigidity of the substituent groups, which weakened the hydrogen bond interactions and thus lowered the thermal decomposition temperature [34]. The initial thermal degradation temperature of OS-PG was nearly identical to that of DDS-PG. Despite a higher grafting of carboxyl groups onto OS-PG, the longer alkenyl chain in DDSA increased steric hindrance between PG molecules, reducing hydrogen bond interactions. The final solid residue of the modified PG was greater than that of the native PG, likely due to the presence of the substituent groups.

## 4. Conclusions

The chain length of alkenyl succinic anhydrides significantly influenced the functionality of hydrophobically modified phytoglycogen (PG), enabling tailored designs for specific applications. OS-PG, exhibiting the highest pH sensitivity and digestive resistance, demonstrates great potential for pH-triggered drug delivery systems, particularly for targeted delivery in intestinal environments. In contrast, DDSA-PG’s superior shear stability and viscosity are ideal for enhancing the texture of processed functional foods, such as low-fat spreads or protein-fortified gels. Additionally, the study establishes a clear correlation between the chain length of modifying anhydrides and the properties of modified PG, offering a valuable framework for selecting PG-based carriers in nutraceutical delivery and food engineering. This work also underscores the potential of ODSA-PG as a stabilizer for hydrophobic bioactives in emulsions. Future research should prioritize encapsulating bioactive models (e.g., curcumin) to evaluate loading efficiency and further validate the biocompatibility of modified PG, which will be crucial for accelerating its translational adoption into commercial applications.

## Figures and Tables

**Table 1:** Degree of substitution (DS), weight-average molecular weight (Mw), radius of gyration (Rz), particle size, polydispersity (PDI), and zeta potential of the samples.

	DS (10^−2^)	Mw (10^7^ g/mol)	Rz (nm)	Particle Size(nm)	PDI	Zeta Potential (mV)
PG	0	1.64 ± 0.02 ^e^	15.85 ± 0.49 ^a^	32.27 ± 0.35 ^a^	0.15 ± 0.00 ^a^	−3.70 ± 0.23 ^d^
3%OS-PG	1.53 ± 0.02 ^c^	1.89 ± 0.01 ^f^	16.75 ± 0.21 ^a^	34.42 ± 0.04 ^b^	0.18 ± 0.01 ^ab^	−11.03 ± 0.45 ^b^
9%OS-PG	4.32 ± 0.08 ^e^	1.86 ± 0.05 ^f^	19.10 ± 0.71 ^b^	35.13 ± 0.06 ^b^	0.19 ± 0.02 ^b^	−17.33 ± 0.39 ^a^
3%DDS-PG	1.07 ± 0.07 ^b^	1.50 ± 0.02 ^c^	16.00 ± 0.13 ^a^	34.80 ± 0.64 ^b^	0.17 ± 0.01 ^ab^	−10.34 ± 0.20 ^b^
9%DDS-PG	2.79 ± 0.02 ^d^	1.44 ± 0.07 ^b^	18.95 ± 0.64 ^b^	36.91 ± 0.61 ^c^	0.19 ± 0.01 ^b^	−16.17 ± 0.55 ^a^
3%ODS-PG	0.75 ± 0.02 ^a^	1.560 ± 0.02 ^d^	15.65 ± 0.34 ^a^	38.42 ± 0.14 ^d^	0.22 ± 0.01 ^c^	−7.26 ± 0.33 ^c^
9%ODS-PG	1.00 ± 0.03 ^b^	1.35 ± 0.03 ^a^	19.75 ± 0.21 ^b^	41.09 ± 0.67 ^e^	0.26 ± 0.01 ^d^	−9.95 ± 1.34 ^b^

Note: Samples of phytoglycogen modified with different anhydrides (OSA, DDSA, and ODSA) at different concentrations (3% and 9%) were named 3%OS-PG, 3%DDS-PG, 3%ODS-PG, 9%OS-PG, 9%DDS-PG, and 9%ODS-PG, respectively. Values were expressed as the mean ± SD (*n* = 3). In the same column, under the same in different samples, significant differences are indicated by different superscript lowercase letters (*p* < 0.05).

**Table 2:** Fit law model and Herschel–Bulkley model parameters of modified PG-based nanoparticle.

	Power Law			Herschel–Bulkley			
	K (Pa·s^n^)	n	R^2^	σ_0_ (Pa)	K (Pa·s^n^)	n	R^2^
PG	0.00525	0.856	0.998	0.00516	0.00405	0.909	0.999
3%OSA-PG	0.00537	0.854	0.998	0.00570	0.00404	0.912	0.999
9%OSA-PG	0.00562	0.862	0.998	0.00652	0.00416	0.925	0.999
3%DDSA-PG	0.00544	0.857	0.998	0.00567	0.00414	0.914	0.999
9%DDSA-PG	0.00780	0.782	0.996	0.0117	0.00460	0.891	0.999

Note: Samples of phytoglycogen modified with different anhydrides (OSA and DDSA) at different concentrations (3% and 9%) were named 3%OS-PG, 3%DDS-PG, 9%OS-PG and 9%DDS-PG, respectively.

**Table 3:** Digestibility and weight loss at different stages in TGA of PG and modified PG.

				Weight Loss at Different Stages			
	RDS	SDS	RS	Stage 1 (%)	Stage 2 (%)	Stage 3 (%)	Solid Residue (%)
PG	69.50 ± 0.25 ^e^	17.89 ± 0.84 ^b^	12.61 ± 1.10 ^a^	7.76 ± 0.18 ^c^	56.11 ± 0.18 ^a^	20.38 ± 0.27 ^e^	15.72 ± 0.32 ^a^
3%OS-PG	46.54 ± 0.46 ^c^	18.81 ± 0.85 ^b^	34.65 ± 1.31 ^c^	6.10 ± 0.08 ^b^	56.78 ± 0.09 ^ab^	18.04 ± 0.10 ^d^	19.06 ± 0.17 ^b^
9%OS-PG	25.45 ± 1.48 ^a^	10.39 ± 1.76 ^a^	64.16 ± 0.27 ^e^	5.38 ± 0.10 ^a^	57.69 ± 0.03 ^b^	9.00 ± 0.40 ^b^	27.86 ± 0.21 ^d^
3%DDS-PG	51.60 ± 0.32 ^d^	17.22 ± 0.97 ^b^	31.18 ± 0.66 ^b^	6.44 ± 0.22 ^b^	61.92 ± 1.00 ^c^	12.73 ± 0.75 ^c^	18.90 ± 0.31 ^b^
9%DDS-PG	33.51 ± 0.66 ^b^	11.10 ± 0.47 ^a^	55.39 ± 1.13 ^d^	5.61 ± 0.08 ^a^	64.57 ± 0.08 ^d^	5.83 ± 0.32 ^a^	23.99 ± 0.47 ^c^

Note: Samples of phytoglycogen modified with different anhydrides (OSA and DDSA) at different concentrations (3% and 9%) were named 3%OS-PG, 3%DDS-PG, 9%OS-PG and 9%DDS-PG, respectively. In the same column, significant differences between different samples are indicated by different superscript lowercase letters (*p* < 0.05).

## Data Availability

The original contributions presented in this study are included in the article. Further inquiries can be directed to the corresponding author.
